# Virtual Group Exercises and Psychological Status among Community-Dwelling Older Adults during the COVID-19 Pandemic—A Feasibility Study

**DOI:** 10.3390/geriatrics6010031

**Published:** 2021-03-22

**Authors:** Amirah Ibrahim, Mei Chan Chong, Selina Khoo, Li Ping Wong, Ivy Chung, Maw Pin Tan

**Affiliations:** 1 Ageing and Age-Associated Disorders Research Group, University of Malaya, Kuala Lumpur 50603, Malaysia; ami.fatin@gmail.com; 2 Department of Nursing Science, Faculty of Medicine, University of Malaya, Kuala Lumpur 50603, Malaysia; mcchong@ummc.edu.my; 3 Centre for Sport and Exercise Sciences, University of Malaya, Kuala Lumpur 50603, Malaysia; selina@um.edu.my; 4 Department of Social and Preventive Medicine, Faculty of Medicine, University of Malaya, Kuala Lumpur 50603, Malaysia; wonglp@ummc.edu.my; 5 Department of Pharmacology, Faculty of Medicine, University of Malaya, Kuala Lumpur 50603, Malaysia; ivychung@ummc.edu.my; 6 Department of Medicine, Faculty of Medicine, University of Malaya, Kuala Lumpur 50603, Malaysia; 7 Department of Medical Sciences, Faculty of Healthcare and Medical Sciences, Sunway University, Selangor 47500, Malaysia

**Keywords:** older adults, physical exercise, virtual exercise, COVID-19

## Abstract

Social isolation, magnified by the restriction of movement order during the COVID-19 pandemic, may lead to negative psychosocial health impacts among community-dwelling older adults. We, therefore, aimed to evaluate recruitment rates, data collection, and group exercises conducted through virtual technology among individuals aged 60 years and over in Malaysia. Participants were recruited from the Promoting Independence in Seniors with Arthritis (PISA) pilot cohort through social media messaging. A four-week course of virtual group exercise was offered. Anxiety and depression were assessed with the Hospital Anxiety and Depression Scale (HADS) during the last attended follow-up of the cohort study (pre-pandemic), pre-intervention, and post-intervention. Exercise adherence was recorded using diaries with daily entries and attendance to the virtual group exercise sessions were also captured electronically daily. The outcomes of interest were changes in anxiety and depression scores from baseline to pre-intervention (pandemic-related) and post-intervention (virtual exercise related). Forty-three individuals were recruited. A significant increase in anxiety scores from baseline to pre-intervention was observed. Comparisons using repeated-measures analysis of variance between those who attendance ≥14 and <14 group exercise sessions revealed no between-within subject differences in depression scores. There was a 23% dropout rate in the post intervention survey and 60.5% of diaries were returned. Virtual group exercises could be conducted among older adults residing in a middle-income country, though recruitment would have been limited to those with internet access.

## 1. Introduction

Social isolation is associated with increased risk of poor health and death, with the magnitude of increase in risk reported to be as high as cigarette smoking [1]. However, in order to curb the spread of the Severe Acute Respiratory Syndrome Coronavirus-2 (SARS-CoV-2), the virus responsible for the current Coronavirus Disease 2019 (COVID-19) pandemic, many countries had resorted to variable degrees of lockdown measures. Even when lockdowns are relaxed, social distancing practices are still advocated as the new norm until a vaccine finally becomes available [2]. In Malaysia, individuals aged 60 years and above make up an estimated 60% of all deaths, further fuelling government recommendations for older persons to stay at home as much as possible, relying on the community to provide for them. The country continues to observe various degrees of restrictions in response to changes in the number of new cases, with the return of the lockdown measures in many states in response to the third wave of the pandemic in Malaysia.

While these measures were effective in controlling viral transmission, they are expected to have a negative impact on the psychosocial health of the community [3,4,5]. Psychological distress was reported between 12 and 52 percent in older adults during this pandemic [6,7]. The heightened risk of severe COVID-19 illness for older persons further increased fear among older people. Therefore, community-dwelling older people living in urban areas stayed at home and became socially isolated as a result of both pandemic response measures as well as anxiety-induced self-imposed isolation. As a result of the mandatory self-isolation, physical activity appears to decline, affecting subjective well-being [8,9]. A large body of evidence supports the benefit of regular physical activities in reducing health risks [10]. We, therefore, conducted a feasibility study to evaluate recruitment, data collection and group exercise intervention delivered through virtual technology among individuals aged 60 years and over in Malaysia, an upper middle-income developing nation in South-East Asia. This feasibility study describes the process of executing the study and to inform the development of the protocol for larger randomized-controlled-trial [11]. This study will therefore provide insights into introducing the use of technology for social activities in the form of group exercise among older adults, a practice previously unfamiliar to them and health care professionals.

## 2. Materials and Methods

### 2.1. Sample Population

**Figure 1:**
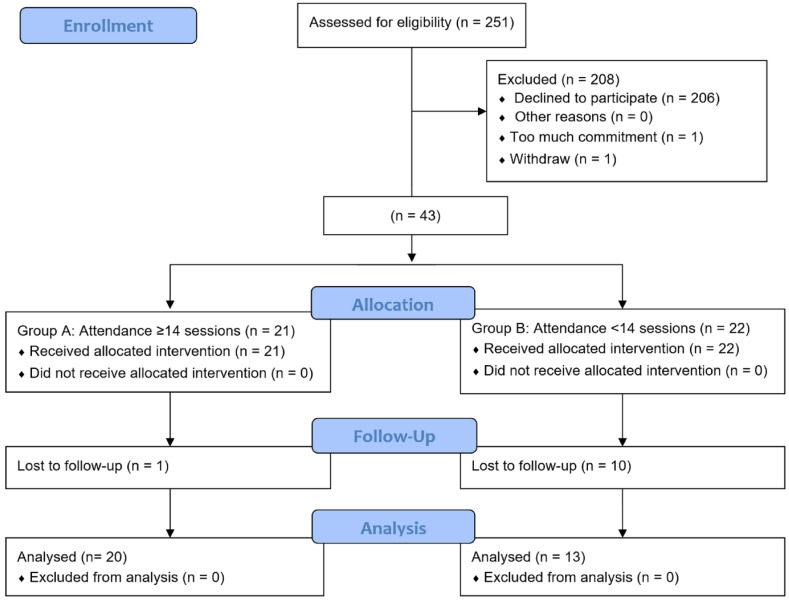
CONSORT flow diagram.

The Caring for Mental Health During the Covid Pandemic (CaMHeP) study was initiated as part of a university-wide initiative to mitigate potential psychological effects of the pandemic. Participants were recruited from a pre-existing pilot longitudinal cohort study, Promoting Independence in our Seniors with Arthritis (PISA) which was recruited in 2015 (Figure 1). A total of 251 individuals aged 55 years and over with and without osteoarthritis were recruited and followed-up annually, with the final follow-up appointment occurring in 2019. Therefore, up to four follow-up visits were made per participant. Ethics approval had been obtained prior and written informed consent was obtained from participants prior to recruitment (UM.TNC2/UMREC-622). Individuals who were unable to perform physical exercise, had ongoing untreated heart conditions, and those unable to participate in telephone or video interviews were excluded.

### 2.2. Recruitment Procedure

The researcher communicated with participants via social media messaging (WhatsApp^TM^, Facebook Inc., Menlo Park, CA, USA), telephone calls and video conferencing (Google Meet^TM^, Google LLC, Moutain View, CA, USA). The WhatsApp^TM^ social media messaging application was used in this study because it is the key networking method and widely used among urban Malaysian older adults [12]. Participants’ information was extracted from the PISA database and those with mobile phone numbers were approached first via a WhatsApp bulk messaging application with text invitation and a short teaser video. Participants who replied to the text or simply replied “yes” were contacted via telephone calls to provide further detailed explanations. Details provided included information on exercises on offer and a link to complete the mental health assessment and Google Meet^TM^ installation. Participants who needed hard copies of the materials were identified and the material was delivered via courier. Participants were included in a conditional Whatsapp^TM^ Group chat, in which only the researcher had access to post to the group. Participants were advised to send message privately to the researcher for any queries.

### 2.3. Measures

Mental well-being was measured at baseline (pre-pandemic), at recruitment to the intervention study, and again at the end of the exercise intervention. Baseline scores were obtained from participants’ last visit recorded between one to two years prior to this study. The pre- and post-intervention scores were assessed between May and June 2020. Additional measures obtained from the PISA dataset were physical activity, social participation, and social network.

#### 2.3.1. Mental Health

Mental well-being was measured using the Hospital Anxiety and Depression Scale (HADS) [13]. HADS was specifically designed for non-psychiatric practice as a self-assessment mood scale. The short time (2 to 5 min) required to complete the scale [14] is an advantage. The scale has 14 items related to anxiety and depression divided equally. The scale scores range from 0 to 21 for each sub-scale, which in this study were analyzed separately. For each sub-scale, a score of 0–7 is regarded as normal, 8 to 10 is suggestive of the presence of either depression or anxiety, and higher than 11 indicates the probable presence of either depression or anxiety [14].

#### 2.3.2. Physical Activity

To measure physical activity level, the International Physical Activity Questionnaire–Short Form (IPAQ-SF) was administered. The IPAQ-SF is a self-administered questionnaire that evaluated physical activity in the last seven days. It comprised seven items that enquired about the presence and time spend performing vigorous activities, performing moderate activities, walking and sitting. The levels of physical activity (low, medium and high) were then determined using a published analysis protocol [15,16].

#### 2.3.3. Social Participation

To measure the occurrence of participation restriction, the Keele’s Assessment of Participation (KAP) was used. KAP is an 11-item self-administered questionnaire. Four levels of restrictions (no restrictions, minimal, moderate and substantial) are then identified available published cut-offs [17].

#### 2.3.4. Social Network

The Lubben Social Network Scale (LSNS-6) was used to evaluate participants’ social network. Total scores ranged from 0 to 30 [18]. Participants with scores below 12 were considered at risk of social isolation.

### 2.4. Intervention

**Figure 2:**
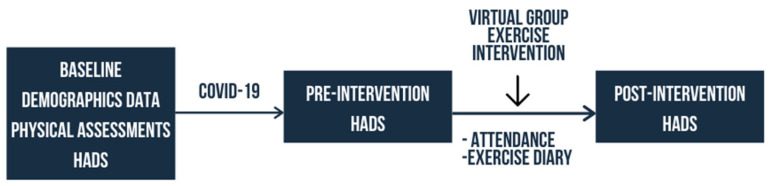
Study framework.

A quasi-experimental study comparing two groups determined through number of attendances to the virtual exercise sessions was conducted (Figure 2). All participants were invited to take part in a four-week course of daily virtual group exercises. Participants with virtual group exercises attendance of 14 or more were included in Group A while those attended fewer than 14 sessions were included in Group B. The cut-off of 14 session was determined using the median attendance level. Exercise adherence was recorded using a daily exercise diary. Both the booklet and virtual exercises focused on muscle strengthening by gradually increasing repetitions, coordination and fine motor training. The study protocol had been registered with the National Malaysian Research Registry (NMRR-20-1629-55960). The four-week virtual exercise was tailor-designed for older persons by the authors and a physiotherapist. Recruited participants were invited to take part in a 30-min daily virtual exercise class. The installing instructions of Google Meet were sent via text and video demonstration. A 2-h trial run or rehearsal session was set up two days before the actual start of the program. The researcher continued to provide technical support from the time of the trial run up to the start of the program and throughout all exercise sessions through the social media messaging group set up for this purpose.

The virtual exercise class started with deep breathing and correction of posture. Exercises for neck, chest, shoulder, upper back, lower back, abdomen, thigh and ankle rotation were conducted in the seated position. Lower back and abdominal strengthening exercise were conducted in the supine position. Hip abduction and adduction, calf and ankle exercises were then conducted in the standing position. Coordination and fine motor training included face muscles exercise, fingers movement exercise, alternating hand exercise, and towel toe curl. Additionally, ankle alphabet exercise were performed to improve flexibility. The sessions ended with deep breathing and a reminder to fill-in their exercise diary. The range of exercises explained above were performed alternately and gradually increments in repetitions which were limited largely by time availability.

Participants who were unable to attend the virtual group exercise were encouraged to conduct individual home-based exercises guided by an electronic exercise booklet and video. The “PISA exercises for older adults” video and booklet had been developed in 2016 as part of a public engagement activity of the original research program. Those who did not participate in virtual exercises were reminded to exercise daily and to fill-in their diary, either via the WhatsApp^TM^ group chat or private messages.

#### Adherence

Adherence to both virtual groups and home-based individual exercises were measured using self-administered exercise diaries with daily entries. This collected information on which days of the week the participant had conducted their exercises. Exercise diaries were either sent out electronically or through couriers as per participant preference. In addition, attendances were taken for each virtual group exercise sessions as an additional measure of adherence.

### 2.5. Data Analysis

Data analysis was performed using the IBM SPSS Statistics 21 and a *p* value of less than 05 was considered statistically significant. Descriptive statistics were presented in frequencies with percentages and mean with standard deviation. Mixed between-within subject ANOVA was used to investigate the effect of time (baseline, pre and post intervention), intervention and interactions between time and groups. Missing data were analyzed using Little’s MCAR test and multiple imputation performed.

## 3. Results

### 3.1. Recruitment

Two hundred and fifty-one PISA participants were approached via the social media messaging application WhatsApp^TM^, of whom 45 agreed to participate with two dropouts before the exercise program started (Figure 1). A 23% drop-out was recorded for the online post-intervention survey after two reminders sent out via WhatsApp^TM^ in group and personal text messages.

### 3.2. Groups Comparison

The two groups were homogenous in years-of-education, gender, ethnicity, marital status, person(s) dwelling with participants, self-rated health condition and health compared to others (*p >* 0.05). The mean age for participants in Group A were significantly higher than those in Group B. Participants in Group B had lower self-rated health compared to others (Table 1).

A paired-samples t-test was conducted to evaluate the impact of the pandemic on anxiety and depression scores of all participants. There was a statistically significant increase in mean anxiety scores from baseline (*M =* 3.5, *SD* = 3.5) to pre-intervention (*M =* 6.02, *SD* = 3.97, *t* (41) = −3.75, *p* = 0.001). No significant mean difference in depression scores from baseline (*M =* 4.98, *SD* = 3.92) to pre-intervention (*M =* 5.57, *SD* = 3.66, *t* (41) = −1.02, *p* = 0.31).

A complete case mixed between-within subjects analysis of variance showed no difference between groups and across time in anxiety scores (Wilks’ Lambda = 0.96, *F* (2, 30) = 0.61, *p =* 0.55, partial eta squared = 0.04), and depression scores (Wilks’ Lambda = 0.89, *F* (2, 30) = 1.80, *p =* 0.18, partial eta squared = 0.11). With multiple imputation on post-intervention anxiety scores, there was a substantial main effect for time (*p <* 0.005) suggesting a significant increase in anxiety scores across time between baseline, pre- and post-intervention. Post-hoc test (Bonferroni) showed significant increase in anxiety scores between baseline anxiety scores and both pre- and post-intervention. There was no significant difference between before and after intervention in anxiety scores (Supplementary Table S1).

**Figure 3:**
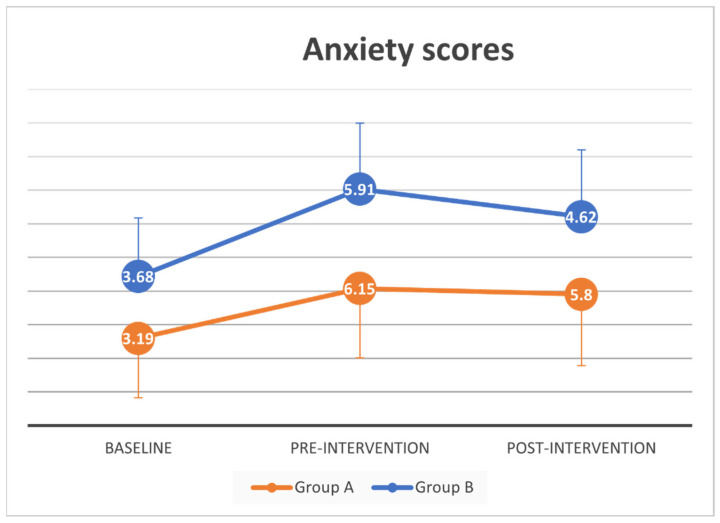
Anxiety scores at baseline, pre and post intervention.

**Figure 4:**
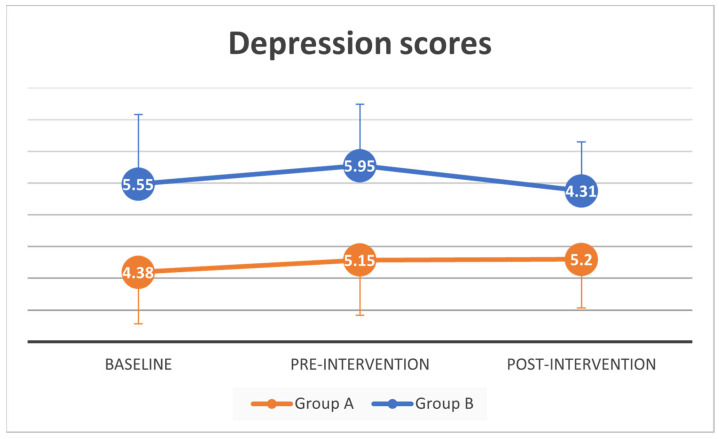
Depression scores at baseline, pre and post intervention.

Depression scores were not significantly different across time or between groups for both original and imputed data analysis. Changes in anxiety and depression scores over time for all participants and the two intervention groups are also summarized in Figure 3 and Figure 4.

### 3.3. Adherence

#### 3.3.1. Attendance

Overall mean attendance was 10.26 (SD = 7.02) sessions. Mean attendance for Group A was 16.43 (SD = 2.16) sessions, minimum attendance was 14 and maximum attendance was 20 sessions. Most participants attended 14 sessions (*n* = 8) and one attended all the 20 sessions. Individuals in Group B had mean attendance of 4.36 (SD = 4.44) sessions.

#### 3.3.2. Exercise Diary

Briefly, 60.5% (*n* = 26) participants returned their exercise diaries. 85.7% (*n* = 18) of participants in Group A returned the exercise diary compared to 36.4% (*n* = 8) from participants in Group B (*p* = 0.002) (Table 2). There was a significant difference in mean attendance between participants in both groups who returned and did not returned the diary *t*(41) = 4.79, *p <* 0.001, two-tailed. No difference in age, years of education, gender, HADS score at baseline, physical activities, co-morbidities, KAP, and LSNS-6 (*p* > 0.05) was found between participants who returned and did not return the diary.

On a sub-group analysis of both groups, there was no significance difference in age, years of education, gender, HADS score at baseline, physical activities, co-morbidities, KAP and LSNS-6 (*p* > 0.05). In Group B, there was a significance mean difference in attendance for participants who returned (*M =* 6.88, *SD =* 4.94) and did not return the diary (*M =* 2.93, *SD =* 3.54) (*t*(20) = 2.18, *p* = 0.041).

Only five out of 26 participants (19.2%) recorded the same amount of attendance in their diary as captured by Google Meet attendance. Further, 20 (76.9%) participants recorded higher number of attendances in their diary compared to captured by Google Meet attendance. A Cohen’s kappa agreement analysis between Google Meet captured attendance and diary attendance for virtual exercises session showed slight agreement, κ = 0.137, *p* = 0.004.

#### 3.3.3. Technical Issues

Of the 45 recruited participants, 10 individuals successfully joined the initial trial run which occurred two days before the start of the program. Text messages (*n* = 9) and phone calls (*n* = 6) were received between the two days on issues including inability to download the application, unable to see or turn on video/audio, forgetting the time of the session, and device memory is full. Queries were attended on an individual basis. A further three trial runs were conducted separately in between the two days for three individuals. Real-time step-by-step demonstration of the web browsing procedure was helpful. Text or voice instructions alone were insufficient. The simultaneous use of two devices (laptop and smart phone) was essential to facilitate successful initiate connection to the virtual meeting platform. Participants were mostly considerate and avoided contacting the researcher in the evenings. The researcher established ground rules and only responded to queries from 7 am until 9 pm. Due to the overwhelming number of calls and texts, a separate number was established. To ensure authenticity, the social media messaging group icon displayed the project logo.

## 4. Discussion

This study was conducted at the peak of the first wave of the COVID-19 pandemic worldwide. Growing anxiety among older people is likely to be linked to the higher mortality rates recorded among people aged 60 years and older from COVID-19 [19,20]. A study conducted in the UK found that 12.8% of participants reporting worse depression scores while 12.3% felt worse on the anxiety scores using the HADS scale [21]. On the contrary, worsening depressive symptom was not observed among our participants. Both studies, nevertheless, clearly demonstrated a worrying trend towards increase in psychological symptoms associated with the COVID-19 pandemic among older adults.

As this study was primarily intended to evaluate the feasibility of employing virtual technology in research within an older population in a developing country, process evaluation is more important than outcomes for which this study was not powered for. There is a perception that older people are unable to use technology and often pester younger family members and acquaintances for help with gadgets. This may be reflected in the low recruitment rate of 45 people out of 251 who responded to social media messaging invitations. Urban older Malaysians have been reported to use social medical text messaging successfully, but are unable to install the application independently [12]. Nevertheless, this study has demonstrated that it is possible to recruit through social media messaging. Consequently, a sample selection bias was unavoidable, as those who responded and participated were able to operate devices independently, indicating that such a strategy does work for at least a segment of the older population in our setting. Prior research work on social media use in research recruitment has centred on young adults or hard-to-reach populations such as those with HIV [22,23,24]. Low accrual rates are in fact expected for social media recruitment and it is recommended that multiple platforms are used.

An acceptable dropout rate was, however, recorded among those who agreed to participant. In face to face exercise intervention studies, dropout rates of 14% to 31.9% have been reported [25,26,27,28] while online dropout rates of 7.5% [29] in studies with sample less than 200 and a large study (*n* = 36,373) recorded 48% non-adherence [30]. Adherence was 93% for a telephone-guided exercise program [31] and 70% for community-based face-to-face programs [32].

Inconsistencies were identified between diary entries and Google Meet^TM^ attendance records. Participants were inclined to fill in diaries during the final day or week of the exercise program, once researcher asked for the diaries to be returned despite daily reminders. The observed differences in self-reported and objective recording of attendance, highlights the need to record attendances, which is facilitated in this study by a software, and highlights potential inaccuracies in self-administered exercise diaries. Technical glitches in the extension software utilized for electronic attendance records were, however, also possible.

The process of preparing the participants for the online program is time consuming which has replaced logistics issues associated with face-to-face programs. The older participants needed time to process new information on the use of online applications to conduct exercise session. Prior to the intervention, only a handful had used other video conferencing platforms for communication, and none had used Google Meet^TM^, which was the platform selected here. Therefore, intensive support from the researcher was required to enable the participants who signed up to eventually successfully join the virtual exercise sessions. Participants were not able to sign on to the video conferencing platforms using instruction provided through social media text messaging alone or recorded voice instructions alone. Instead support using telephone instructions and video calls with social media applications were used. Nevertheless, once the older persons had mastered the procedures required, they were able to continue to log in to the platform using the link provided for the rest of the study. Prior pre-pandemic efforts at engaging older persons in telehealth using videoconferencing yielded mixed results [33]. Movement restrictions incurred throughout the pandemic and the need to shield our older population has, however, limited the older person’s choice, and therefore potentially altered their determination to engage through videoconferencing. Participants were also able to self-administer questionnaires through an online platform which was deliberately kept as short as possible to enhance completion rates. Experience from face-to-face data collection found that older persons often required a longer time and some assistance to complete questionnaires [34]. A qualitative interview would be appropriate to further identify technical difficulties encountered by participants, however, face-to-face interviews were not possible due to movement restrictions and physical distancing rules, and virtual interviews would pose additional challenges beyond the scope of this study.

This study is limited by the non-randomized allocation to groups. It was a pragmatic decision, since the priority was to test the virtual assessment and interventions, and potential communication issues associated with virtual contact precluded the possibility of detailed explanations that are necessary before randomization can proceed. Allocation of groups to the participants may hinder the motivation for those not selected for virtual exercises. Therefore, the decision was to allow recruitment through convenience sampling and for individuals to select their preferred mode of exercise. However, this resulted in contamination between the groups, because everyone has the links to access the virtual exercise groups. This suggests that individual randomization in future such studies may experience the same issues, and hence cluster randomization with safeguards against contamination will be necessary. Ultimately, the study was able to demonstrate the feasibility of conducting virtual interventions and data collection in a developing nation setting where the older population may have limited information technology literacy, as well as highlight potential limitations in the recruitment, conduct, and representativeness of such a study. Nevertheless, during a pandemic when older adults would otherwise be completely excluded due to the need to physical distance, shield, and obey lockdown measures, conducting online surveys and virtual interventions will at least allow some inclusion.

## 5. Conclusions

Our study had demonstrated that it was possible to recruit, collect data and administer virtual group exercises among individuals aged 60 years and over in a developing nation, though the overall feasibility was limited by a selection bias of those who responded to social media advertisements. The delivery of the intervention requires investment in training and extensive technical support through a variety of methods. While limitations exist with regards to recruitment and treatment allocation, it is still important to include older adults in clinical research using virtual communications, to ensure that older adults are not completely excluded from ongoing studies.

## Figures and Tables

**Table 1:** Demographic characteristics of participants in Group A and Group B.

Variable		Mean ± Standard Deviation				*p*-Value
		Group A (*n* = 21) (14 or >14 sessions)		Group B (*n* = 22) (<14 sessions)		
Age (years)		65.1 ± 4.81		61.8 ± 5.15		Df = 41 *t =* 2.16 *p =* 0.037
Years of education		15.79 ± 2.42		14.48 ± 2.92		Df = 38 *t =* 1.54 *p =* 0.132
Attendance to online sessions		12.43 ± 2.16		4.36 ± 4.44		Df = 41 *t =* 11.4 *p <* 0.001
Variable	Sub-variable	**Group A**		**Group B**		
		*n*	*%*	*n*	*%*	
Gender	Male Female	4 17	19 81	6 15	28.6 71.4	*p =* 0.717
Ethnicity	Malay Chinese Indian Others	3 17 0 1	14.3 81 0 4.8	2 15 5 0	9.1 68.2 22.7 0	*p =* 0.542
Marital status	Single Married Widowed Partner Divorced	1 19 1 0	4.8 90.5 4.8 0	3 15 2 2	13.6 68.2 9.1 9.1	*p =* 0.155
Dwelling	Independent Spouse Children Parent	0 17 3 1	0 81 14.3 4.8	7 11 4 0	31.8 50 18.2 0	*p =* 0.070
Health condition (self-rated)	Poor Fair Good Very Good	0 2 14 5	0 9.5 66.7 23.8	2 6 12 2	9.1 27.3 54.5 9.1	*p =* 0.085
Health compared to others	Poor Fair Good Very Good Excellent	0 0 11 9 1	0 0 52.4 42.9 4.8	1 9 7 4 1	4.5 40.9 31.8 18.2 4.5	*p =* 0.002

**Table 2:** Attendance summary.

Variables	All	Group A (*n* = 21) (14 or > 14 sessions)	Group B (*n* = 22) (<14 sessions)
Number of returned diaries	26	18	8
Total exercises sessions attended	666	464	202
Total exercise attended per person	25.6	25.8	25.3
Total virtual group exercises	441	345	96
Total virtual group exercises per person	10.3	19.2	12
Total individual home exercises	225	119	106
Total individual home exercises per person	8.7	6.6	13.3

## Data Availability

The data presented in this study are available on request from the corresponding author. The data are not publicly available due to ethical and privacy requirement.

## Supplementary Materials

The following are available online at https://www.mdpi.com/2308-3417/6/1/31/s1, Table S1: Comparison of anxiety and depression scores at baseline, before and after intervention between groups.
