# Supplementary material for: Virtual Group Exercises and Psychological Status among Community-Dwelling Older Adults during the COVID-19 Pandemic—A Feasibility Study
Source: Geriatrics (Basel). 2021 Mar 22;6(1):31. doi: 10.3390/geriatrics6010031 (PMC8005958; doi:10.3390/geriatrics6010031)
Supplement: Supplementary file 1 [file geriatrics-06-00031-s001.pdf]

Table S1. Comparison of anxiety and depression scores at baseline, before and after intervention between groups.

Domains	Group A (14 or >14 sessions)	Group B (<14 sessions)	*p*-Value
	Mean (standard deviation)	Mean (standard deviation)	
	Baseline		
Anxiety	3.19 (3.54)	3.68 (3.48)	0.649
Depression	4.38 (3.25)	5.55 (4.38)	
	Before intervention		
Anxiety	6.15 (4.12)	5.91 (3.93)	0.847
Depression	5.15 (3.47)	5.95 (3.86)	
	After intervention		
Anxiety	5.80 (4.24)	4.62 (3.99)	0.428
Anxiety (pooled data)	5.77	5.51	0.853
Depression	5.20 (3.07)	4.31 (3.07)	0.422
Depression (pooled data)	5.17	4.57	0.554
